# Current Research on Theory and Practice of Digital Libraries: Best Papers from TPDL 2019 & 2020

**DOI:** 10.1007/s00799-022-00322-5

**Published:** 2022-02-28

**Authors:** Trond Aalberg, Fabien Duchateau, Mark Hall, Tanja Merc̆un, Thomas Risse

**Affiliations:** 1 Oslomet, Oslo, Norway and NTNU, Trondheim, Norway; 2 University Lyon, UCBL, CNRS, INSA Lyon, LIRIS, UMR5205, F-69622 Villeurbanne, France; 3 The Open University, Milton Keynes, UK; 4 University of Ljubljana, Ljubljana, Slovenia; 5 Goethe University Frankfurt, University Library J. C. Senckenberg, Frankfurt, Germany

**Keywords:** Digital libraries, TPDL conference, Special issue

## Abstract

This volume presents a special issue on selected papers from the 2019 & 2020 editions of the International Conference on Theory and Practice of Digital Libraries (TPDL). They cover different research areas within Digital Libraries, from Ontology and Linked Data to quality in Web Archives and Topic Detection. We first provide a brief overview of both TPDL editions, and we introduce the selected papers.

## Overview of TPDL 2019 & 2020 editions

The TPDL conference constitutes a leading scientific forum that brings together researchers, developers, content providers, and practitioners in the field of Digital Libraries. Digital libraries and repositories store, manage, represent, and disseminate rich and heterogeneous data that are often of enormous cultural, scientific, educational, artistic, and social value. Serving as digital ecosystems for empowering researchers and practitioners they provide unparalleled opportunities for novel knowledge extraction and discovery. New applications raise novel challenges that can only be addressed in an interdisciplinary community of researchers and practitioners from various disciplines including the Digital Humanities, Information Sciences, and others.

The 23$^{\mathrm{rd}}$ edition of TPDL took place at the Oslo Metropolitan University, Norway, on September 9–12, 2019. The general theme was *connecting with communities* to reflect the need for digital libraries to interact with researchers and practitioners for effective data utilization, management, and exploitation. The proceedings contain 17 long research papers, 11 short research papers, and 18 poster and demonstration papers [2]. The 24$^{\mathrm{th}}$ edition of TPDL was special for two reasons. First, it should have taken place at the Université de Lyon, France, from August 25 to 28, 2020, but due to the COVID-19 pandemic, the event was organized online from August 25 to 27, 2020 [5]. Secondly, it was held jointly with two conferences in Information Systems, namely the 24$^{\mathrm{th}}$ European Conference on Advances in Databases and Information Systems (ADBIS) and the 16$^{\mathrm{th}}$ French EDA days on Business Intelligence & Big Data. The proceedings feature 14 long research papers and 4 short papers, whose topics span from knowledge graphs, linked data and ontology design to user studies, digital cultural heritage, and research data management [4].

Following the tradition of previous TPDL editions [3, 6], the Program and General chairs decided to prepare a volume consisting of extended versions of the best papers from TPDL 2019 & 2020. This gives invited authors an opportunity to add details or new contributions and to describe additional experiments or studies, but also to consider perspectives suggested by reviewers. For the Digital Library community, such a volume is useful to establish the progress made so far in our field through mature works as well as to identify future research directions.

## Presentation of Selected Papers

Papers accepted in both editions of TPDL had already been thoroughly reviewed by three reviewers and one senior meta-reviewer. Additionally, all papers were discussed during a meeting of the Program and General chairs for producing the list of TPDL accepted papers. From the pool of papers accepted in TPDL 2019 & 2020, the General and the Program Committee Chairs nominated ten papers based on their reviewing scores: three from TPDL 2019 and seven from TPDL 2020. Authors of these papers were invited to extend their original paper by at least 30% in a four-months period. Nine new versions were submitted. As required by IJDL’s policy, these extended papers went through another round of reviews by at least three reviewers, supported by a senior reviewer. Finally, 6 papers were accepted for publication in this special issue. Below is a short description of these papers.

The paper *An Extended Analysis of the Persistence of Persistent Identifiers of the Scholarly Web* by Martin Klein and Lyudmila Balakireva investigates the notion of persistence of DOIs by analyzing their resolution on the web. Digital Object Identifiers (DOIs) are common standard to persistently identify resources. The DOI concept is based on the assumption that mappings between the resources and the DOIs are updated when the resource location is changing. The authors show that persistence is in reality not guaranteed. Instead content providers respond differently to varying request methods and network environments and even change their response to requests against the same DOI. The paper presents quantitative analysis results and aims informing the scholarly communication community about this disconcerting lack of consistency.

Brenda Reyes Ayala presents *Correspondence as the Primary Measure of Information Quality for Web Archives: A Human-Centered Grounded Theory Study*, a grounded theory of quality specifically for web archives. She analyzed support tickets submitted by clients of the Internet Archive’s Archive-It (AIT). Therefore, the theory is human-centered and grounded on the perception of users and creators on the quality of web archives. The resulting theory compromises the dimensions correspondence, relevance, and archivability. Web archivists and cultural heritage institutions will benefit from the clarified notion of quality in a web archive.

The next paper by Arthur Brack, Anett Hoppe, Markus Stocker, Sören Auer, and Ralph Ewerth on *Analysing the Requirements for an Open Research Knowledge Graph: Use Cases, Quality Requirements and Construction Strategies* tackles the issue of exploring and comparing scientific research literature in a semantic way using an Open Research Knowledge Graph (ORKG). They first describe the typical tasks of a researcher (use cases) that can be supported by an ORKG, such as finding related work or obtaining a deep understanding of a paper. Minimal requirements (in terms of granularity or completeness for instance) are defined for each use case. Next, manual, semi-automatic, and automatic approaches for building an ORKG are surveyed, both for designing ontologies and for populating the graph. This article thus provides a broad overview of the different requirements for an ORKG, and it fosters future research on this emerging topic.

The *VeTo+: improved expert set expansion in academia* article by Serafeim Chatzopoulos, Thanasis Vergoulis, Theodore Dalamagas, and Christos Tryfonopoulos extends their work on identifying experts in a given field, for example, in order to identify new reviewers for a conference. Rather than relying on data containing explicit links between experts and topics, the authors identify potential experts by considering the venues the experts publish in and the topics attached to the publications. The extended algorithm presented here builds on this by enabling dynamic weighting of the two factors and filtering of the considered publication venues. The second is of particular interest to interdisciplinary venues, such as TPDL or JCDL, where except for the interdisciplinary venue, there is often little publishing overlap between experts.

The following paper *Multi-Label Classification of Legislative Contents with Hierarchical Label Attention Networks* by Danielle Caled, Mário J. Silva, Bruno Martins, and Miguel Won deals with the issue of annotating legislative documents using the EuroVoc hierarchical thesaurus. Contrary to existing annotation methods, authors propose a deep learning model to take into account the three levels of the thesaurus rather than predicting for a single level. In order to evaluate this approach, more than 200,000 legal documents in Portuguese, which have been classified according to EuroVoc, are integrated into the dataset EUR-Lex PT. This article finally highlights recurring challenges for automatic annotation of large collection of documents, such as the long tail label distribution which degrades accuracy.

Angelo Salatino, Francesco Osborne, and Enrico Motta’s paper *CSO Classifier 3.0: A Scalable Unsupervised Method for Classifying Documents in Terms of Research Topics* extends their work presented at TPDL 2019 by introducing an improved design of the Computer Science Ontology (CSO) Classifier for automatic classification of research papers according to the CSO and presenting novel mechanisms for detecting outlier topics. To measure the performance of their solutions, the authors created a gold standard using 70 documents, manually annotated by domain experts. The paper also describes how the classifier has been adopted by other researchers since 2019 and how the classifier could be adapted for classifying with other knowledge organization systems as the code is freely available to the wider research community.

To conclude, we believe that this volume of selected Digital Libraries papers reflects the ongoing work and topic trends in our field. We hope that our readers will find them insightful, and we invite them to take note about the next editions of TPDL (online in 2021 [1] and in Padova in 2022).
